# CareConnect: An Implementation Pilot Study of a Participatory Telecare Model in Long-Term Care Facilities

**DOI:** 10.3390/healthcare14030335

**Published:** 2026-01-28

**Authors:** Miriam Hertwig, Franziska Göttgens, Susanne Rademacher, Manfred Vieweg, Torsten Nyhsen, Johanna Dorn, Sandra Dohmen, Tim-Philipp Simon, Patrick Jansen, Andreas Braun, Joanna Müller-Funogea, David Kluwig, Amir Yazdi, Jörg Christian Brokmann

**Affiliations:** 1 Center for Clinical Acute and Emergency Medicine, University Hospital RWTH Aachen, 52074 Aachen, Germany; 2 Haus Hörn, 52074 Aachen, Germany; 3 SKM Rothe Erde, 52068 Aachen, Germany; 4 MVZ Aachen Campus Practice, 52074 Aachen, Germany; 5 Clinic for Surgical Intensive Care Medicine and Intermediate Care, University Hospital RWTH Aachen, 52074 Aachen, Germanytsimon@ukaachen.de (T.-P.S.); 6 Department of Operative Dentistry, Periodontology, and Preventive Dentistry, University Hospital RWTH Aachen, 52074 Aachen, Germany; pjansen@ukaachen.de (P.J.);; 7 Clinic for Dermatology and Allergology, University Hospital RWTH Aachen, 52074 Aachen, Germany

**Keywords:** telecare, participatory design, implementation science, interprofessional collaboration, nursing homes

## Abstract

**Highlights:**

**What are the main findings?**

- Telecare was rated feasible for certain clinical use cases in nursing homes.
- Participatory implementation and early involvement facilitated acceptance and use among nurses.

**What is the implication of the main finding?**

- Sustainable telecare adoption requires interoperable infrastructure, ongoing workforce training, context-sensitivity, and clear reimbursement structures.
- Co-design and iterative strategies appear essential for successful uptake of telecare in long-term care facilities.

**Abstract:**

**Background**: Digital transformation in healthcare has advanced rapidly in hospitals and primary care, while long-term care facilities have often lagged behind. In nursing homes, nurses play a central role in coordinating care and accessing medical expertise, yet digital tools to support these tasks remain inconsistently implemented. The *CareConnect* study, funded under the German Model Program for Telecare (§ 125a SGB XI), aimed to develop and implement a multiprofessional telecare system tailored to nursing home care. **Objective**: This implementation study examined the feasibility, acceptability, and early adoption of a multiprofessional telecare system in nursing homes, focusing on implementation processes, contextual influences, and facilitators and barriers to integration into routine nursing workflows. **Methods**: A participatory implementation design was employed over 15 months (June 2024–August 2025), involving a university hospital, two nursing homes (NHs), and four medical practices in an urban region in Germany. The telecare intervention consisted of scheduled video-based teleconsultations and interdisciplinary case discussions supported by diagnostic devices (e.g., otoscopes, dermatoscopes, ECGs). The implementation strategy followed the Standards for Reporting Implementation Studies (StaRI) and was informed by the Consolidated Framework for Implementation Research (CFIR). Data sources included telecare documentation, nurse surveys, researcher observations, and structured feedback discussions. Quantitative and qualitative data were analyzed descriptively and triangulated to assess implementation outcomes and mechanisms. **Results**: A total of 152 documented telecare contacts were conducted with 69 participating residents. Most interactions occurred with general practitioners (48.7%) and dermatologists (23%). Across all contacts, in 79% of cases, there was no need for an in-person visit or transportation. Physicians rated most cases as suitable for digital management, as indicated by a mean of 4.09 (SD = 1.00) on a 5-point Likert scale. Nurses reported improved communication, time savings, and enhanced technical and diagnostic skills. Key challenges included delayed technical integration, interoperability issues, and varying interpretations of data protection requirements across facilities. **Conclusions**: This pilot study suggests that telecare can be feasibly introduced and accepted in nursing home settings when implemented through context-sensitive, participatory strategies. Implementation science approaches are essential for understanding how telecare can be sustainably embedded into routine nursing home practice.

## 1. Introduction

Worldwide, health systems are facing increasing structural strain in long-term care delivery. This strain results from demographic change, population aging, rising care complexity, and persistent workforce shortages across nursing and medical professions [1]. In nursing homes, these pressures are compounded by high care demands, limited staff capacity, and challenges in accessing timely medical expertise for residents with complex or acute health needs [2,3]. In this context, nurses play a central role as primary providers and coordinators of day-to-day care, frequently serving as the first point of clinical assessment and decision-making [4]. Together, these factors threaten the continuity, quality, and safety of care delivery in long-term care settings.

In response to these challenges, digitalization is increasingly discussed as one potential instrument to support care delivery and coordination. Digital health technologies such as telecare and teleconsultations may facilitate access to medical expertise, improve interprofessional communication, and support clinical decision-making [5,6]. Additionally, in the context of nursing homes, the literature reports that telehealth may reduce avoidable organizational effort (e.g., transfers) and help overcome challenges resulting from the COVID-19 pandemic [7]. While digital health technologies are already increasingly established in hospitals and outpatient care, nursing homes have often been marginalized in digital transformation processes. Reasons for this lie in the differences between long-term care facilities and acute care settings in care trajectories, organizational routines, staffing patterns, regulatory frameworks, and reimbursement structures. Care is predominantly continuous and relationship-based rather than episodic, and digital interventions must support ongoing decision-making rather than isolated acute events. Nevertheless, more and more studies are emerging that pilot and implement telehealth or telecare in nursing homes [7,8].

However, digitalization is not a solution in itself to the multidimensional challenges facing health systems, particularly in nursing homes. Evidence shows that poorly integrated technologies can increase workload or disrupt workflows when organizational, regulatory, and infrastructural conditions are not adequately addressed [9,10]. Additionally, the COVID-19 pandemic revealed potential but also highlighted problems occurring with digital technologies in fragile care systems: telecare and teleconsultations enabled continuity of care under conditions of restricted mobility and infection control requirements, but staffing shortages and digital infrastructure, especially in nursing homes, limited their effectiveness [11]. Systematic reviews consistently identify barriers to telecare implementation in nursing homes, including insufficient technical infrastructure, limited interoperability with existing documentation systems, unclear reimbursement mechanisms, and uncertainties related to data protection and legal responsibilities [9,10,12]. Moreover, digital interventions in long-term care cannot be reduced to technological feasibility. They require alignment with nursing workflows, clear role definitions, and active involvement of care professionals [13,14,15]. These experiences underline the need for context-sensitive, carefully implemented digital interventions rather than technology-driven, one-size-fits-all approaches. Implementation science frameworks such as the Consolidated Framework for Implementation Research (CFIR) [16] emphasize that successful adoption of complex interventions depends on contextual factors, stakeholder engagement, capacity building, and iterative adaptation. Similarly, the Standards for Reporting Implementation Studies (StaRI) [17] recommend a clear distinction between the intervention and the implementation strategy to enhance transparency and replicability.

Despite a growing body of research on telecare in nursing homes, important gaps remain in how such interventions are studied and reported. Existing studies frequently emphasize clinical outcomes or technical feasibility, while offering limited insight into the implementation processes through which telecare systems are developed, adapted, and embedded into routine nursing home practice. In particular, the application of implementation science frameworks to systematically examine contextual determinants, stakeholder engagement, and mechanisms of change remains limited. In addition, participatory co-design approaches are often mentioned but rarely analyzed in depth with respect to their influence on intervention specification and workflow integration.

The CareConnect study was designed to address this gap. In two nursing homes in Germany, a multiprofessional telecare system was collaboratively developed and implemented to support nursing and medical decision-making in nursing home care. Rather than evaluating clinical effectiveness, this pilot study focuses on the implementation process, examining feasibility, acceptability, early patterns of adoption, and the facilitators, barriers, and mechanisms influencing integration into everyday practice. Using a process evaluation informed by the Consolidated Framework for Implementation Research (CFIR) this study contributes empirically grounded insights into the conditions under which telecare can be embedded in long-term care settings.

## 2. Materials and Methods

### 2.1. Study Design

A participatory implementation design was applied over 15 months (June 2024–August 2025) in an urban region in Germany, involving a university hospital, two nursing homes (NH A and NH B), and four medical practices. The NHs were a convenience sample and recruited via direct contact and were selected to reflect contrasting baseline conditions with regard to size and digital infrastructure.

The study followed the Standards for Reporting Implementation Studies (*StaRI*), distinguishing between the telecare intervention and the implementation strategy [17]. The implementation strategy was conceptually informed by the Consolidated Framework for Implementation Research (CFIR) [16] and comprised participatory co-design, training, iterative testing, and structured feedback loops. The telecare intervention consisted of scheduled video consultations and interdisciplinary case discussions supported by diagnostic devices. Further specifications of the intervention were part of the iterative co-design process.

### 2.2. Setting and Contextual Characteristics (Outer and Inner Setting)

The two NHs involved (A and B) were characterized by different contextual factors which were documented prior to implementation. 

NH A was a smaller facility caring for 56 people, with an emphasis on dementia care, located in the central part of a German city where the study took place. NH A was adequately covered by care due to the presence of a general practitioner located nearby. Additionally, other medical specialists, such as dermatologists and dentists, were available for nursing home visits, but at varying frequencies.

NH B was considerably larger, caring for approximately 171 residents, located in the same city, but not centrally. It offered two special wards with intensive long-term care for people in a persistent vegetative state or the need for mechanical ventilation. This nursing home also had a very good coverage of care by several general practitioners and some specialists, like neurologists and an anesthesiologist, for the ventilated patients. NH B had a preexisting Wi-Fi infrastructure and a nursing documentation system known for its interoperability options. These contextual factors informed the scope of feasible telecare use cases, scheduling decisions, and technical integration priorities.

### 2.3. Telecare Intervention

The telecare intervention consisted of video-based teleconsultations and interdisciplinary case discussions between nursing staff and external medical professionals using a certified video platform. Telecare contacts were supported by diagnostic devices depending on the clinical question (e.g., high-resolution wound camera, vital signs monitor, digital stethoscope, otoscope, dermatoscope, ECG, and dental/oral diagnostic tools). Telecare was implemented primarily as scheduled sessions, with defined professional contacts and time slots. This concept and the final schedule and participating disciplines were determined through participatory co-design and feasibility discussions (see Section 2.4).

### 2.4. Implementation Strategy and Conceptualization Process

**Figure 1:**
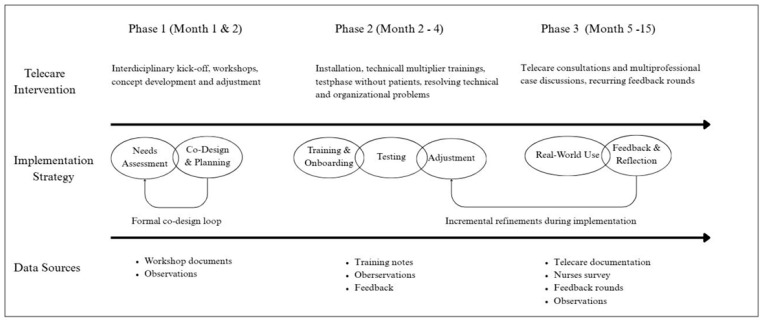
Phased implementation of the CareConnect telecare system, distinguishing the telecare intervention from the implementation strategy.

The implementation strategy comprised three iterative phases, each linked to anticipated mechanisms of change (engagement, capacity building, workflow integration, iterative refinement), which were assessed in the process evaluation (Figure 1):

1. Needs assessment and co-design: At the beginning, three interdisciplinary workshops were held to identify care needs, communication barriers, and expectations regarding telecare and its use cases. The mechanism of change employed was stakeholder engagement, expected to increase acceptability and workflow fit. Based on the workshop findings, the project team developed an initial telecare concept tailored to the organizational routines and technical capabilities of both NHs (see Section 3.1.).
2. Training and technical onboarding: Following the co-design phase, all participating nurses received hands-on training on the use of the telecare system (including the certified video consultation software and additional diagnostic devices like otoscopes, dermatoscopes, and ECGs). The training sessions functioned as multiplier trainings, enabling internal dissemination of skills. Test runs were conducted to ensure technical readiness and to familiarize staff with telecare workflows. Capacity building was expected to increase digital competence and reduce uncertainty.
3. Implementation and continuous feedback: During the 11-month implementation phase, teleconsultations and interdisciplinary case discussions were conducted. The process was accompanied by three structured feedback rounds, in which nursing staff provided input on usability, technical challenges, perceived benefits, and workflow obstacles. Through this iterative refinement, the implementation strategy was adapted, where applicable, in response to feedback to achieve the expected outcome of progressively integrating telecare into daily routines.

#### 2.4.1. Conceptualization Loop and Refinement Logic

These phases resulted in a single co-design loop (workshop-based identification of desired contacts, followed by feasibility clarification with potential telecare partners, and, lastly, revision of the concept into a scheduled model). During the implementation phase, continuous refinements, informed by three structured feedback rounds and ongoing observation of telecare use in practice, resulted in minor adjustments. These refinements did not alter the core intervention logic but entailed minor adaptations, including adjustments to scheduling, clarification of workflows, and resolution of technical or organizational issues.

#### 2.4.2. Workshops

A kick-off workshop of 3 h included nursing staff from both nursing homes, participating physicians (hospital and outpatient), and IT/technical experts (n = 25). Two institution-specific workshops each 90 min were held with nursing staff (NH A: n = 6; NH B: n = 8). The workshops aimed to test preselected equipment, identify priority clinical scenarios and interprofessional coordination needs, define feasible telecare contacts, and surface anticipated barriers (technical, workflow, legal/data protection, staffing). Decisions on concepts or adjustments of concepts were made by consensus. Workshop data consisted of structured field notes taken by the research team during discussions and equipment testing, and documented outputs from the workshop (e.g., lists of desired contacts/use cases, scheduling preferences, and noted constraints). Workshop materials were included in the qualitative document corpus and analyzed using systematic document analysis focusing on needs, proposed use cases, constraints, and implementation requirements. Workshop outputs were consolidated by two researchers into a draft concept, which was then consented to in follow-up calls with NH representatives and participating departments.

### 2.5. Data Sources

Data were derived from four different sources:

- Telecare documentation: The standardized documentation in project-specific spreadsheets completed by physicians after each telecare contact included date of contact, reason for contact, diagnostic instruments used, outcome/treatment decision, and need for re-contact. Furthermore, physicians’ evaluation of the digital suitability of the contact and the technical reliability was measured using a 5-point Likert scale.
- Structured online nurse survey: This anonymous survey was offered to nurses after every telecare contact from May to August 2025. This survey captured the total frequency of contacting professionals and the reasons for the contacts. Nurses’ assessment of the usefulness of the contact, the extent to which the problem could be resolved, and the technical reliability was measured using a 5-point Likert scale.
- Researcher observations: Field notes were taken during workshops, training sessions, test runs, and feedback meetings.
- Semi-structured participant feedback was obtained during regular round-table discussions and short debriefings during the implementation phase.

These complementary data sources provided the empirical basis for analyzing usage patterns, progress in implementation, and user experiences during the implementation phase.

### 2.6. Process Evaluation

The process evaluation examined whether the implementation strategy operated through mechanisms of change (engagement, capacity building, workflow integration, iterative refinement) and how contextual factors shaped adoption (infrastructure, staffing, routines, technical integration). CFIR domains and indicators were operationalized as shown in Table 1.

### 2.7. Analytical Framework

Quantitative Analysis: Structured variables such as the number and characteristics of telecare contacts, the reasons for consultation, the diagnostic tools used, and assessments of digital suitability were extracted from the project-specific spreadsheets and analyzed descriptively (absolute and relative frequencies, means, ranges where applicable) to characterize usage patterns and the perceived appropriateness of telecare. No inferential statistics were performed.

Qualitative Analysis: Data about the implementation strategy, workshops and process evaluation were examined using systematic document analysis and analyzed using descriptive coding. Coding was performed by one researcher. To enhance trustworthiness, CFIR mapping decisions and emerging themes were discussed in regular team meetings and cross-checked against quantitative patterns (triangulation). Coding focused on observable barriers and facilitators, workflow adaptations, technical integration issues, and perceived effects. Findings were organized along CFIR domains and implementation mechanisms (Table 1).

Quantitative and qualitative findings were combined at the interpretive level.

### 2.8. Ethical Considerations

Ethical approval was obtained from the Ethics Committee of University Hospital RWTH Aachen (EK 24-290). Informed consent was obtained from all participants involved.

## 3. Results

Results are presented in the order of the implementation process’s sequential phases. Quantitative usage data are integrated with qualitative observations and participant feedback to illustrate implementation outcomes (e.g., feasibility, acceptability, adoption, digital suitability) and process mechanisms (e.g., stakeholder engagement, capacity building, iterative refinement, and workflow integration). Results are reported in alignment with the CFIR domains and indicators operationalized in Table 1, with qualitative and quantitative findings mapped to intervention characteristics, inner and outer setting factors, individual characteristics, and implementation processes.

### 3.1. Needs Assessment and Co-Design

#### Outcomes of Participatory Co-Design: Telecare Concept Development

The participatory workshops constituted the intervention development method; the resulting telecare concepts are presented to document how the telecare intervention was specified prior to implementation. During the initial interdisciplinary kick-off workshop (n = 25), participants tested the preselected telecare equipment, discussed potential use cases, and reflected on care challenges (CFIR: Inner Setting, Process–Engagement). Although general practitioner coverage was described as broadly adequate, participants highlighted gaps in timely access to specialist care—particularly for complex situations in NH B—and difficulties obtaining medical assessments shortly before weekends or during evenings, especially for suspected infections. Participants also discussed spontaneous telecare contacts; however, scheduled consultations were perceived as more predictable and compatible with nursing home routines.

In institution-specific workshops (NH A: n = 6; NH B: n = 8), local needs and barriers were further specified, including concerns about Wi-Fi stability, variable staff confidence in handling devices, and the anticipated availability and willingness of external professionals to participate. These workshop outputs were synthesized into an initial telecare concept (Table 2). In Table 2, “yes” indicates that participants identified the contact as a priority and feasible from the nursing home perspective; “probably” indicates a desired contact with anticipated feasibility constraints (e.g., partner availability, workflow fit).

Following co-design, the research team approached hospital departments to explore the feasibility of the proposed contacts, while nursing homes contacted their general practitioners. Feasibility discussions indicated that not all specialties were willing or able to provide telecare, mainly due to concerns about clinical appropriateness and workflow compatibility. As a result, the telecare model was refined into a scheduled format with defined time slots to increase predictability and routine integration. The final intervention specification implemented during the 11-month phase is shown in Table 3.

### 3.2. Training/Onboarding

In accordance with the intervention specification, the required hardware and software were installed in both nursing homes. The technical provider conducted five structured training sessions across both sites, enabling selected staff to act as multipliers in accordance with the Medical Devices Act. Additional internal sessions focused on practical procedures (e.g., preparing wound images, uploading medication plans, assembling diagnostic devices). Physicians provided device-specific instruction for the safe use of otoscopes, wound cameras, and oral diagnostic tools.

In total, 37 nurses completed formal training (NH A: n = 14; NH B: n = 23). A subsequent testing phase without residents revealed substantial technical challenges, including non-functional interfaces with the nursing documentation system, failed image transfers, and calibration issues. These issues were addressed with the technical provider, and routine telecare use commenced in study month five.

### 3.3. Qualitative Findings: Implementation and Continuous Feedback

During the implementation phase, teleconsultations and interdisciplinary case discussions were conducted according to the schedule outlined in Table 3. Three structured round-table discussions supported ongoing reflective monitoring (CFIR: Process–Reflection & Adoption). Across observation notes and feedback rounds, recurring patterns related to communication quality, workflow integration, and confidence in telecare use were identified. Participants repeatedly described respectful and solution-oriented interactions with physicians, which were perceived as facilitating interprofessional collaboration (required during kick-off, reported in all feedback rounds) (CFIR: Process–Engagement):

“*The communication with the physicians is always respectful, and we were able to solve problematic cases jointly*” (NH A).

Participants also reported perceived reductions in organizational effort in 2 of 3 feedback rounds (e.g., time savings) in situations where resident transport could be avoided, reflecting improved workflow compatibility within the nursing home setting (CFIR: Inner Setting):

“*It would cost us a lot more time to arrange transport and accompaniment for our residents to a practice than the telecare contact does*” (NH B).

Over time, nurses described increasing confidence and familiarity with the telecare system and associated diagnostic devices (not during training but in 2 of 3 feedback rounds). Rather than indicating objective skill gains, these accounts reflect self-reported confidence development and perceived competence in routine use (CFIR: Characteristics of Individuals):

“*By now I know how the system works, it’s no problem for us to teach the physicians [who are not used to the system yet]*” (NH B).

“*I feel like a little expert now using the oral scanner and the dentist is always available via video and will directly tell me if I missed a spot [in the mouth]*” (NH A).

At the same time, recurrent barriers were identified across data sources. These included staff absences (in 2 of three feedback rounds), difficulties securing substitutes for scheduled sessions, and technical disruptions such as login errors, server interruptions, or failed data transfers (in all feedback rounds) (CFIR: Process–Execution):

“*Right now, there are public holidays, this is why I don’t find other colleagues who can attend the [telecare] contact*” (NH A).

These challenges were consistently reported during feedback rounds and documented in observation notes.

Across both NHs, telecare was perceived as particularly suitable for acute care situations (e.g., respiratory infections, wounds, medication questions), dermatological follow-ups, and dental assessments (CFIR: Intervention Characteristics). In intensive care settings, telecare supported clinical discussions about secretion management, therapy progress, ventilation adjustments, and assessments of weaning potential.

### 3.4. Quantitative Results

A total of 152 telecare cases with the predefined contacts were documented for 69 residents, with 31 residents receiving multiple assessments. Three unplanned additional contacts were provided by an emergency physician specialized in trauma care for two patients. The majority of interactions involved the general practitioner offering infectious disease consultations, including a wound specialist (48.7%), and the second most interactions involved dermatologists (23%). Across all cases, the high-resolution wound camera was the most used diagnostic tool (40.7%), followed by the vital signs monitor (28.9%) and the stethoscope (14.4%). 79% of telecare cases were resolved without an on-site visit (although telecare control or on-site secondary control may have been required), whereas 21% required resident transport (after discussion of the problem, transport was necessary to resolve it). 82% of all concerns could be managed entirely digitally (there was no need for further on-site contact). Table 4 presents the results of telecare contacts by specialist and reason for contact. Across all documented telecare contacts, physicians rated the general suitability of the cases for digital management with a mean of 4.09 (SD = 1.00) on a 5-point Likert scale, indicating that most situations were perceived as appropriate for telecare. Technical performance was evaluated favorably by physicians (mean = 4.21, SD = 1.22).

**Figure 2:**
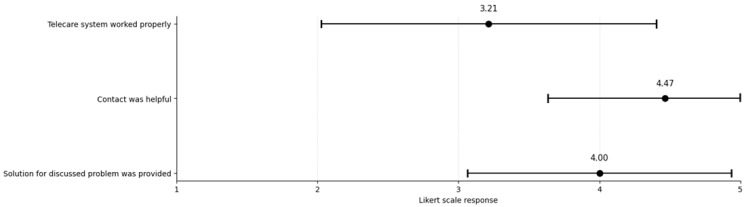
Nurse responses regarding technical feasibility, usefulness of telecare, and solutions provided during contacts. Points represent mean values; horizontal error bars indicate ±1 standard deviation. The Likert scale ranged from 1 = strongly disagree to 5 = strongly agree. n = 23 nurses.

The nurse survey, with a response rate of 41.8% (n = 23 responses), supported these observations. 65.5% of telecare contacts evaluated were referred for consultations with general physicians or infectious disease specialists. The usefulness of the contacts and the extent to which the problem could be resolved consistently received high ratings on the Likert scale. In contrast, the technical functioning of the system was judged more variably compared to the physicians’ ratings. Figure 2 displays the average nurse ratings, along with their standard deviations, for the three assessed aspects.

**Figure 3:**
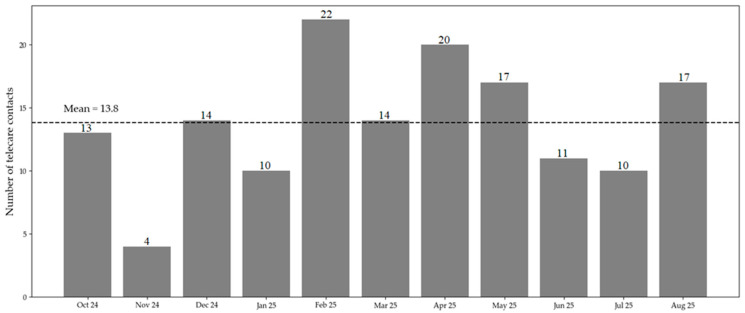
Monthly number of telecare contacts during the 11-month implementation phase. Numbers above bars indicate absolute counts per month. The dashed line represents the mean number of monthly telecare contacts (mean = 13.8).

The usage patterns of the telecare system over time are shown in Figure 3. There were fewer contacts in November 2024 and June and July 2025. Additional researchers’ notes revealed a staff shortage due to a wave of illness and public holidays. Those months were challenging for the NHs in maintaining routine care, and additional tasks, such as telecare, could not be prioritized during this period.

Convergence across data sources was observed for several implementation outcomes. High physician ratings of digital suitability and technical reliability were consistent with nurses’ high ratings of usefulness and problem resolution. In contrast, greater variability in nurses’ ratings of technical functioning aligned with observation notes and feedback discussions documenting intermittent technical disruptions. Furthermore, reduced telecare activity, as identified in usage data for November 2024 and June–July 2025, coincided with staffing shortages reported during feedback rounds and documented in researchers’ field notes, indicating the influence of organizational capacity on adoption.

### 3.5. Facilitators and Barriers

The facilitators and barriers reported below correspond to the CFIR domains and process indicators defined in Table 1 and are derived from triangulation of observation notes, feedback rounds, and usage data.

Several factors supported the implementation of telecare in both NHs. Early involvement of nursing staff during the co-design phase emerged as a central facilitator, contributing to a sense of ownership and alignment between telecare workflows and daily care routines (CFIR: Process–Engagement; Inner Setting). Participants described the co-design process as enabling realistic expectations regarding telecare use and integration. Hands-on training and the availability of ongoing technical support were repeatedly identified as supporting factors for telecare adoption (CFIR: Characteristics of Individuals; Process–Execution). Over time, nurses reported increasing confidence in using the system, which facilitated its adoption and peer-to-peer support, including assistance for physicians unfamiliar with the technology. Regular feedback rounds provided a structured opportunity to identify and address emerging issues, enabling incremental adaptations to workflows and schedules (CFIR: Process–Reflection & Adoption). Participants emphasized that respectful, solution-oriented communication with physicians fostered a positive work environment and sustained engagement. In addition, nurses reported high acceptance of telecare among residents. Residents were described as curious and willing to engage with video-based consultations and perceived the care provided by connected medical professionals as adequate and reassuring (CFIR: Intervention Characteristics).

Barriers to implementation were consistently reported across observation notes and feedback discussions. Technical limitations, such as unreliable electronic prescription transmission to pharmacies and intermittent system disruptions, led to delays in care processes (CFIR: Intervention Characteristics; Inner Setting). Legal and reimbursement-related constraints were identified as external barriers. Lower reimbursement rates for video consultations compared with on-site visits, and challenges related to processing health insurance cards, limited consistent participation by some external physicians (CFIR: Outer Setting). Organizational and structural factors also influenced scalability. Telecare implementation proved highly dependent on local conditions, particularly staffing availability. Scheduled telecare sessions functioned well within the participating nursing homes but were not always easily transferable to external practices with differing workflows (CFIR: Inner Setting; Process–Execution). Finally, interoperability issues between information systems and heterogeneous interpretations of data protection requirements across institutions further complicated implementation and limited seamless integration into existing care infrastructures (CFIR: Outer Setting; Inner Setting).

#### Conditions for Long-Term Implementation

This section summarizes sustainability requirements as articulated by participants. Future needs were identified through end-of-study feedback discussions with nursing staff and nursing home management and were consistent with barriers observed during implementation. Participants emphasized internal requirements for sustainability, including stable infrastructure, continuous training/support, interoperable documentation interfaces, and integration of telecare competencies into professional education and onboarding routines. Externally, participants identified the absence of clear reimbursement mechanisms and funding for licenses/maintenance as key barriers. They also highlighted the need for consistent regulatory guidance (documentation, consent, data protection) and system-level interoperability across nursing homes, practices, hospitals, and pharmacies. Political and strategic support was considered necessary to embed telecare into routine long-term care structures, alongside continued evaluation of process quality and user experiences.

## 4. Discussion

This study examined the early implementation of a telecare system in two long-term care facilities and identified the factors influencing its feasibility, acceptability, and integration into daily care. By applying implementation science frameworks, the study contributes process-level insights into the mechanisms and contextual factors shaping telecare adoption in long-term care settings.

Across data sources, telecare was perceived as acceptable and could be operationalized in routine workflows when supported by the implementation strategy. Quantitative documentation indicated that a substantial proportion of cases were managed without on-site visits and that physicians rated many contacts as suitable for digital management. At the same time, variability in nurses’ ratings of technical functioning and repeated reports of interruptions indicate that feasibility was conditional and depended on technical stability and local capacity.

Overall, the findings suggest that telecare can be feasibly introduced and perceived as acceptable by nursing staff when embedded within a structured, participatory implementation strategy. Still, successful telecare implementation depends on a set of process- and context-related factors, which aligns with existing evidence [18,19]. The results are consistent with prior studies that emphasize the iterative nature of digital adoption and the importance of active stakeholder participation, training, and technical adaptation [15,20,21]. Similarly to earlier implementation research in long-term care, the present study highlights that feasibility and acceptance are not inherent properties of the technology itself, but emerge from the interaction between technical design, organizational context, and implementation processes.

### 4.1. Principal Findings in Relation to the Implementation Strategy

Interpreted through the Consolidated Framework for Implementation Research (CFIR), several domains emerged as particularly influential in shaping implementation outcomes:

- Intervention Characteristics: Telecare was perceived as useful for specific clinical questions (e.g., infections, wounds, dermatology follow-up, dental assessment). These perceptions align with the notion that implementation success depends on perceived relative advantage and task–technology fit. However, perceived “time savings” and “improved collaboration” were self-reported and should be interpreted as acceptability signals rather than objective workflow impact.
- Inner Setting: Organizational readiness and infrastructure (Wi-Fi availability, interoperability with documentation systems, and local scheduling routines) strongly influenced day-to-day execution. Differences between the two NHs illustrate why a “one-size-fits-all” telecare approach is unlikely to work: implementation required adaptation to local routines and technical ecosystems.
- Characteristics of Individuals: Nurses’ confidence with telecare and devices appeared to increase over time, supported by hands-on training and repeated use. These observations indicate perceived competence development; they do not establish objective skill gains but suggest that implementation strategies should explicitly include capacity building, refreshers, and onboarding pathways.
- Process: Participatory co-design and structured feedback rounds supported engagement and enabled iterative refinement. The observation that later-onboarded staff expressed greater uncertainty aligns with Normalization Process Theory, which emphasizes that early shared understanding and collective commitment are strong predictors of sustained adoption [22]. Staff who did not participate in the initial co-design and training phases appeared to have fewer opportunities to develop shared meaning and confidence, suggesting that implementation strategies should explicitly address staff turnover and onboarding to prevent fragmentation over time.
- Outer Setting: Reimbursement constraints, legal/administrative requirements, and cross-organizational interoperability barriers remained major determinants and were largely not modifiable by local implementation efforts. This finding reinforces that NH telecare implementation is shaped by system-level conditions and that sustainability depends on policy and infrastructure beyond the NHs themselves.

The study thus reinforces the distinction between what can be achieved through local implementation efforts and what requires broader policy and regulatory change.

### 4.2. Implications

Despite its pilot nature, the study yields several implications for practice and policy. At the organizational level, telecare implementation in nursing homes should prioritize participatory design, structured training, and continuous feedback mechanisms to support alignment with nursing workflows. Embedding telecare competencies into nursing education and routine onboarding processes may help mitigate challenges related to staff turnover.

At the system level, sustainable implementation will require supportive reimbursement structures, interoperable digital infrastructures, and clear regulatory guidance. Furthermore, interoperable data interfaces between nursing documentation systems, outpatient and inpatient electronic records, and pharmacy systems should be legally mandated to eliminate double documentation and data silos. National initiatives, such as Germany’s Digitalization Strategy [23] and the newly obligatory use of the Telematics Infrastructure (TI) [24], as well as the emerging European Health Data Space (EHDS) [25], support this. However, NHs require practical guidance and financial support to implement these requirements effectively.

### 4.3. Limitations

Several limitations should be acknowledged when interpreting the study’s findings. First, this pilot study involved only two long-term facilities in a single region and lacked a control group, which limits the generalizability of the findings and precludes causal conclusions. Furthermore, there is a risk of selection bias in the participating nursing homes due to the convenience sample. Facilities that opt to participate may differ systematically from non-participating nursing homes, for example, with respect to organizational capacity, openness to innovation, or prior experience with interprofessional collaboration. These factors may have contributed to more favorable implementation conditions than would be expected in the broader long-term care sector. Both nursing homes reported comparatively favorable relationships with multiple physicians and other healthcare professionals. Such conditions are not universally present, particularly in rural or underserved regions, where access to physicians and continuity of medical care may be more limited. Accordingly, challenges related to physician availability and coordination may be underestimated in the present study.

Additionally, the high level of provider involvement observed may be attributable to the strong engagement of a well-connected attending university hospital. This degree of institutional support may not be available in non-academic settings, potentially limiting the transferability of the findings to facilities without comparable professional networks.

Due to funding constraints, the implementation period was restricted to a very short implementation window of 11 months, which precludes conclusions about long-term adoption, sustainability, and integration into routine practice, as implementation dynamics change over time. Also, the study relied in part on self-reported data from nurses and physicians, which introduces the possibility of recall and social desirability bias.

The telecare intervention was developed through a single formal co-design loop, followed by incremental refinements during implementation. While multiple full co-design cycles might have enabled a broader exploration of alternative intervention models, the chosen approach reflects the pragmatic constraints of a pilot implementation study embedded in routine care. The combination of a single structured co-design phase with continuous feedback-driven adaptation enabled timely implementation while maintaining responsiveness to real-world challenges. However, future studies could benefit from multiple formal co-design iterations, particularly to explore alternative scheduling models, broader stakeholder involvement (e.g., additional specialties or payers), and enhanced generalizability across different long-term care contexts.

Finally, the observed implementation process and the identified barriers and facilitators may therefore differ in settings with lower medical support, reduced digital readiness, or more constrained resources, further limiting the transferability of the observed implementation experiences.

### 4.4. Future Developments

Future developments in telecare for nursing homes are likely to be shaped by structural changes in the nursing workforce, regulatory frameworks, and digital health infrastructure, as well as by ongoing research. In Germany and other European countries, the ongoing academization of nursing (oriented to international standards) and the expansion of advanced and specialized nursing roles are expected to strengthen nurses’ involvement in clinical decision-making and interprofessional collaboration [26,27]. These developments may increase both the demand for and the effective use of telecare as a tool to support nursing-led coordination of care, rather than to replace professional judgment. At the same time, legal and technological developments may reduce some of the external barriers observed in this study. If reimbursement mechanisms, interoperability standards, and data protection regulations become more consistent across care sectors, telecare may become easier to integrate into routine long-term care practice.

From a research perspective, future studies should build on the present findings by examining long-term sustainability, scalability across diverse nursing home contexts, and potential impacts on workflows, care coordination, and resource use. Multi-site studies with longer follow-up periods and comparative implementation strategies will be essential to better understand under which conditions telecare can become a stable component of routine long-term care delivery.

## 5. Conclusions

This pilot study suggests that telecare can be feasibly introduced and perceived as acceptable within nursing home workflows when supported by a participatory and iterative implementation strategy. The study’s main contribution is a process-level account showing that adoption depended on stakeholder engagement, capacity building, and context-sensitive workflow integration, while persistent barriers were driven by system-level reimbursement and interoperability constraints. Telecare should therefore be considered a supportive instrument for nursing staff to facilitate access to medical expertise and coordination, not a standalone solution to structural challenges in long-term care. Longer-term and multi-site research is needed to determine sustainability, scalability, and objective impacts on workflows and outcomes.

## Figures and Tables

**Table 1:** Operationalization of CFIR domains and process outcomes.

CFIR Domain	Operational Definition	Dara Source	Examples
Intervention characteristics	Perceived suitability of telecare for nursing home care tasks	Telecare documentation; physician ratings; nurse survey	Likert-scale ratings of suitability for telecare; proportion of cases managed digitally
Outer Setting	Regulatory, reimbursement, and inter-organizational conditions influencing telecare use	Feedback rounds; observations; participant statements; literature	Reported reimbursement barriers; data protection uncertainties
Inner Setting	Organizational readiness, infrastructure, and workflow compatibility within nursing homes	Context description; workshops; observations	scheduling adherence; integration with documentation systems
Characteristics of Individuals	Digital competence, confidence, and role perceptions of nursing staff	Workshops; Training records; nurse survey; observations	Training participation; self-reported confidence; ability to guide physicians
Process-Engagement	Degree of stakeholder involvement in co-design and implementation	Workshop participation; observations	active contribution
Process-Execution	Practical conduct of telecare sessions in routine care	Telecare documentation; feedback rounds	Completion of scheduled sessions; technical disruptions
ProcessReflection & Adoption	Iterative refinement based on structured feedback	Feedback rounds; observations	Adjustments to schedules, workflows, or technical setup

**Table 2:** First Draft Telecare Concept. It documents site-specific intervention specifications derived through co-design and feasibility refinement.

Desired Telecare Contacts	Nursing Home A	Nursing Home B
Infectious Disease Consultations *	yes	
Wound specialist	yes	probably
Dermatology	yes	
Intensive Care	no	yes
Dentist	yes	no
Neurologist	yes	
Opthalmology	yes	
Ear-Nose-Throat Medicine	yes	
General Practitioner	yes	
Anaesthesiologist	no	yes
Holiday Consultations	yes	
Emergency Department Nurses	probably	

* including wound consultations with a specialized nurse.

**Table 3:** Final Concept Telecare. The Final schedule reflects feasibility agreements with participating partners and local workflow constraints.

Telecare Contacts	Nursing Home A	Nursing Home B
Infectious Disease Consultations *	Wednesday 1 pm	Friday 1 pm
Dermatology	Wednesday 2 pm	Wednesday 1 pm
Intensive Care	Thursday, 2 pm every 2 weeks	-
Dentist	-	Thursday, 10 am every 3 weeks
General Practitioner	Tuesday	Once a week
Anaesthesiologist	Once a week	-
Emergency Department Nurses	Wednesday until 7 pm	
Holiday Consultations	Official Holidays 4 h/day	

* Provided by a general practice not involved in the nursing homes’ care until study start, located next to the university hospital, offering also wound consultations with a specialized nurse.

**Table 4:** Results from telecare documentation.

Telecare Contact	Total Cases	Reason for Contact (% of All Cases)	Managed Completely Digital, % of Each Contact Reason	Reason Suitable for Complete Digital Contact, Mean Likert Scale (SD *)
Infectious disease consultations *	74	Respiratory symptoms (35.1%)	77%	4.28 (0.83)
		Wounds/pressure ulcers (29.7%)	89%	4.16 (0.69)
		Pain (6.8%)	75%	4.0 (0.82)
Dermatology	35	Eczema/dermatitis (28.6%)	100%	4.73 (0.47)
		Pressure ulcers (20%)	100%	4.43 (0.53)
Intensive Care	28	Prurigo (17.1%)	83.4%	3.67 (1.37)
		Therapy progress assessment (46.4%)	69%	4.0 (1.0)
		Assessment of weaning potential (25%)	71.4%	4.67 (0.52)
Dentist	15	Acute deterioration (14.2%)	75%	4.25 (0.96)
		Control (46%)	100%	4.22 (0.83)
		Oral cavity assessment (20%)	66.7%	3.0 (0.0)
		Dental assessment (13%)	100%	3.0 (0.0)
Emergency department	3	Surgical wounds (100%)	66.6%	4.0 (1.73)
General Practitioners	Missing **			

The Likert scale ranged from 1 = not suitable at all to 5 = fully suitable. * SD: standard deviation; ** missing data of outpatient general practitioners due to practice routine and documentation in a separate software.

## Data Availability

The data presented in this study are available on request from the corresponding author due to privacy concerns.

## Abbreviations

The following abbreviations are used in this manuscript:

CFIR	Consolidated Framework for Implementation Research
DNT	Digital nursing technologies
ECG	Electrocardiogram
EHDS	European Health Data Space
GKV	German: Gesetzliche Krankenversicherung
NH	Nursing Home
SGB	German: Sozialgesetzbuch
TI	Telematics Infrastructure
WHO	World Health Organization
